# 

**Published:** 1968-04

**Authors:** 


					1 "
VO \
: $
April 1968
INDEX ?
INPUT
INDEXER: M
DATS;
DEPTH:
NON-DEPTH: V
RUSH:
SELECTIV!
HffltX
INCORPORATING THE MEDICAL JOURNAL OF THE SOUTH WEST
83 (i and ii) No 307

				

